# Rare association of granulomatosis with polyangiitis with an underdiagnosed spondyloarthritis effectively treated with rituximab: A case-report

**DOI:** 10.1097/MD.0000000000041366

**Published:** 2025-01-31

**Authors:** Thomas Escoda, Aurélie Dehaene, Laetitia Velardocchio, Arnaud Deveze, Benjamin Terrier, Laurent Chiche

**Affiliations:** a Service de Médecine Interne, Hôpital Européen, Marseille, France; b Service de Radiologie, Hôpital Européen, Marseille, France; c Service de Pneumologie, Hôpital Européen, Marseille, France; d Service d’Oto-Rhino-Laryngologie, Chirurgie de la Face et du Cou, Clinique Clairval, Marseille, France; e Service de Médecine Interne, Hôpital Cochin, Paris, France.

**Keywords:** antineutrophil cytoplasmic antibody, granulomatosis with polyangiitis, spondyloarthritis, vasculitis

## Abstract

**Rationale:**

Associations of autoimmune diseases are rare but interesting and challenging situations from a diagnostic, pathophysiological, and therapeutic point of view. This article studies a rare association of autoimmune diseases by discussing the pathophysiological hypotheses and an original therapeutic management. The coexistence of antineutrophil cytoplasmic antibody-associated vasculitis and spondyloarthritis has rarely been described.

**Patient concerns:**

We present a patient with inflammatory back pain, stiffness, and enthesopathies followed by pulmonary and ear, nose and throat granulomatous involvement.

**Diagnoses:**

A combination of spondyloarthritis and granulomatosis with polyangiitis, with spinal, enthesopathic, pulmonary, and ear, nose and throat involvement.

**Interventions and outcomes:**

Effective treatment with rituximab both on spondyloarthritis and vasculitis.

**Lessons:**

We discuss the pathogenic, diagnostic, and therapeutic implications of this rare but intriguing association between these 2 inflammatory conditions.

## 1. Introduction

According to the Chapel Hill Consensus Conference nomenclature, small vessel necrotizing vasculitides correspond to antineutrophil cytoplasmic antibody (ANCA)-associated vasculitis (AAV), which includes granulomatosis with polyangiitis (GPA), microscopic polyangiitis and eosinophilic granulomatosis with polyangiitis (EGPA).^[1]^ These diseases are characterized by a pauci-immune necrotizing inflammation of small blood vessels, endothelial injury and tissue damage, and patients with AAV usually present with multisystemic disease that can be severe with serious organ damage, or even life-threatening, although a less severe presentation can also occur. GPA and microscopic polyangiitis preferentially affect the upper (rhinosinusitis, chondritis in GPA classically) and lower (intra-alveolar hemorrhage and diffuse interstitial pneumonia) respiratory tract and kidneys, but all organs can potentially be affected. EGPA is characterized by severe asthma, rhinosinusitis, blood and tissue eosinophilia and vasculitis manifestations (myocarditis, vascular purpura, and peripheral neuropathy).^[2]^ Osteoarticular involvement in AAV is not specific and is often included into the constitutional symptoms of the disease (such as fever, fatigue, anorexia, and weight loss) and is typically described as inflammatory myalgia and arthralgia, or even arthritis of the peripheral joints.^[3]^

The association of AAV with other autoimmune or inflammatory diseases has been described, particularly with rheumatoid arthritis, Sjögren’s syndrome, systemic sclerosis and systemic lupus erythematosus and also inflammatory bowel diseases.^[4–6]^ However, the association of AAV with spondyloarthritis has rarely been described in the literature.

Here, we describe the case of a patient with undifferentiated spondyloarthritis that had evolved over several years who subsequently presented with GPA with pulmonary and ear, nose and throat (ENT) involvement and for whom anti-CD20 (rituximab) led to the remission of both inflammatory conditions. The patient has given his authorization to the publication of this clinical case after informed consent.

## 2. Case presentation

**Figure 1.**
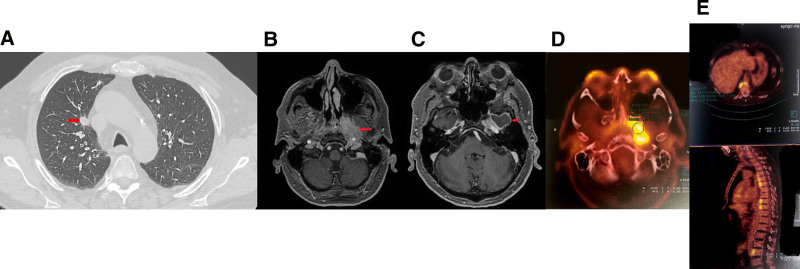
Pretreatment imaging assessment: (A) thoracic computed tomography (CT)-scan showing a speculated pulmonary nodule (red narrow); (B, C) cranial magnetic resonance imaging (MRI) showing a mass infiltrating the left infratemporal fossa (B) and pachymeningitis (C); (D, E) positron emission tomography (PET) scan showing hyperfixation of the ear, nose and throat (ENT) tumor (D) and the anterior longitudinal vertebral ligament (E).

Our case was a 59-year-old male patient with a history of splenectomy and a vertebral fracture after a road accident 30 years previously, active smoking and daily alcohol consumption. For several years, he had suffered from inflammatory rheumatism with nocturnal lumbar pain with stiffness, and enthesopathies, especially at the calcaneus level and the Achilles tendon which had not been diagnosed and for which the patient was taking nonsteroidal anti-inflammatory drugs. At the end of 2022, given the worsening of the pain and the presence of a biological inflammatory syndrome, magnetic resonance imaging (MRI) of the ankle and right foot was performed and revealed Achilles tendinopathy and enthesopathy with calcaneal erosion and plantar aponeurosis consistent with underdiagnosed undifferentiated spondyloarthritis. A second MRI of the spine showed hypersignals of the antero-inferior edge of vertebra T11 and the antero-superior vertebral corner of L2. The patient did not have any digestive symptoms suggestive of inflammatory bowel disease. Due to deterioration in his general health status and active smoking, a thoracic computed tomography (CT) scan was performed and spiculated and multilobulated nodules and masses were identified on his lung parenchyma, the largest of which measured 27 mm (Fig. 1A).

A biopsy of these nodules was performed and histological analysis revealed significant inflammatory changes in his pulmonary parenchyma with aspects of hemorrhagic macrophage alveolitis. There were significant fibrohyaline changes and pseudo-granulomatous histiocytic areas with the presence of pseudo-abscessed noncaseating necrosis and adjacent hyaline fibrosis. No giant cell or eosinophilic infiltrates were found. The diagnosis of malignancy was ruled out.

Laboratory findings revealed: hemoglobin 14.6 g/dL, total white blood cells 14 g/L, neutrophils 8.5 g/L, lymphocytes 3.64 g/L, platelets 483 g/L, C-reactive protein 80 mg/L, increased gamma-glutamyl transferase up to 3-folds normal values, serum creatinine 56 µmol/L (estimated glomerular filtration rate 107 mL/min), normal angiotensin-converting enzyme, positive rheumatoid factor (69 U/mL), and negative antinuclear antibodies. Serum protein electrophoresis showed inflammatory syndrome with a normal level of gamma globulins. HLA-B27 was negative.

A few months later, he then developed left V cranial nerve neuralgia with neuropathic pain and paresthesia on the left side of the face. MRI of the face showed left infratemporal fossa infiltration measuring 36 mm in diameter extending medially to the parapharyngeal space with perineural infiltration along the V3 nerve and pachymeningeal enhancement around it (Fig. 1B and C). An 18-FDG positron emission tomography scan revealed hypermetabolism of this left parapharyngeal pseudo-tumoral lesion, hypermetabolism of pulmonary nodular opacities and condensations, and signs of spondyloarthropathy with clear enthesopathy of the anterior longitudinal ligament in front of 6 vertebrae and bilateral sternoclavicular inflammatory arthropathy (Fig. 1D and E). Screening for ANCA was positive with proteinase 3 specificity. A diagnosis of GPA with ENT (pseudo-tumor) and pulmonary involvement confirmed after careful reviewing of the granulomatous and vasculitis aspect on previously performed lung histology. A diagnosis of undifferentiated spondyloarthritis was retained based on the presence of Achilles tendon enthesitis and a history of inflammatory back pain, in the absence of additional evidence of alternative diagnoses (SAPHO syndrome, psoriasis or inflammatory bowel disease).

**Figure 2.**
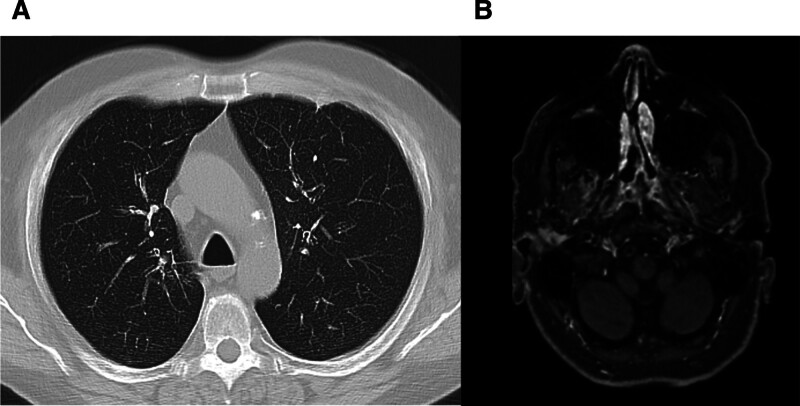
Posttreatment imaging assessment: (A) thoracic CT-scan showing the disappearance of the pulmonary nodule; (B) cranial MRI showing clear regression of the ENT lesion.

Treatment with oral glucocorticoids (1 mg/kg/d) and rituximab (2 infusions of 1000 mg) was initiated and was effective for both pulmonary and ENT lesions as well as enthesopathic and spinal pain, with complete regression of the biologic inflammatory syndrome (Fig. 2).

## 3. Discussion

To our knowledge, only 5 other cases of spondyloarthritis associated with AAV have been described in the literature (Table 1).^[4,7–10]^ These were either GPA, EGPA or anti-myeloperoxidase-associated AAV and the diagnosis of the 2 diseases was either simultaneous or one predominated over the other for several years. Although only a small number of cases have been described, the clinical picture of the 2 diseases seemed typical and the spondyloarthropathies could also be associated with classical extra-articular involvement such as inflammatory bowel disease or psoriasis.

**Table 1:** Characteristics of reported patients with AS and AAV overlap syndrome.

Sex/Ref	Age (AS)	Age (AAV)	Overlap syndrome	AS features	Time between AS and AAV	AAV features	Treatment	Outcome
M (our case)	59	59	US/GPA	Axial spine involvement, enthesopathy, sternoclavicular arthritis	Simultaneous	Pulmonary nodules, cavum pseudo-tumor, PR3+	CTC, RTX	NA
M ^[7]^	?	63	AS/EGPA	Sacroileitis, HLA-B27 +	?	Eosinophilic pneumonia, sinusitis	CTC for EGPA	Good
M ^[4]^	49	49	AS/GPA	IBP, sacroileitis, HLA-B27+	Simultaneous	GN, DAH, ILD, ENT, PN, bronchial stenosis, skin, fever	CTC, CYC, AZA, MMF, PLEX, renal replacement therapy	Died
F ^[8]^	?	65	AS/anti-MPO AAV	IBP	AS before AAV	Large vessel vasculitis, aortitis	CTC (MP), RTX for AAV	Good
F ^[9]^	66	55	Psoriatic arthritis/GPA	Peripheral erosive arthritis, dactylitis, tenosynovitis	11 years GPA before psoriatic arthritis	ENT (rhinitis), pneumonia, PR3+	CTC (MP), CYC, AZA for GPA MTX (for psoriatic arthritis)	Good
F ^[10]^	48	53	CD/AS/psoriasis/GPA	Axial, enthesopathic and peripheral involvement	5 years AS before GPA	Chondritis, GN, fever, pulmonary nodule, PR3+	Anti-TNF, sulfasalazine for AS CTC (MP), RTX	good

AAV = antineutrophil cytoplasmic antibody-associated vasculitis, AS = ankylosing spondylitis, AZA = azathioprine, CD = Crohn disease, CTC = corticosteroids, CYC = cyclophosphamide, DAH = diffuse alveolar hemorrhage, EGPA = eosinophilic granulomatosis with polyangiitis, ENT = ear, nose and throat, GN = glomerulonephritis, GPA = granulomatosis with polyangiitis, IBP = inflammatory back pain, ILD = interstitial lung disease, MMF = mycophenolate mofetil, MP = methylprednisolone, MPA = micropolyangiitis, MPO = myeloperoxidase, NA = not applicable, PLEX = plasmatic exchange, PN = peripheral neuropathy, RTX = rituximab, TNF = tumor necrosis factor, US = undifferentiated spondyloarthritis.

As this disease association is rare we could hypothesize that our patient presented only a single disease, namely GPA with enthesopathic manifestations. It is possible that damage specific to GPA, particularly at the axial or peripheral joint level, could mimic spondyloarthritis, as has already been reported.^[11]^ This atypical enthesopathic involvement in GPA could correspond on an articular level to the digestive manifestations mimicking a chronic inflammatory bowel disease already described in AAV.^[5]^ In the case of our patient, the significant delay between the occurrence of symptoms specific to each disease and the specificity of the clinical and paraclinical disorders argue in favor of the coexistence of the 2 diseases. Then, the question can be raised as to whether these 2 diseases occur together by chance or not. Common physiopathological characteristics exist between these 2 diseases, notably the involvement of a Th17 response, which seems to have a pivotal role both in spondyloarthritis and in AAV. Th17 cells promote the priming and recruitment of neutrophils, facilitate auto-antibody production and have direct, detrimental effects on parenchymal cells at the site of inflammation.^[12,13]^ In GPA, it was shown that patients with active disease had higher levels of IL-17 than those in remission which could suggest a role for treatment targeting IL-17 in this disease.^[14,15]^

Therapeutic management of our patient was challenging due to the association of the 2 inflammatory diseases, particularly GPA, where the presence of a pseudo-tumoural granulomatous involvement is often difficult to treat. According to French recommendations, treatment of these granulomatous masses is based on glucocorticoids combined with immunosuppressive therapy. According to data from a retrospective study in 59 patients with an orbital mass, the response rate was 52% with cyclophosphamide versus 91% with rituximab.^[16,17]^ The effectiveness of rituximab in spondyloarthritis has already been described in the literature. An open-label trial reported data from 20 patients with active ankylosing spondylitis (AS) (10 of whom were anti-TNF naïve) treated with rituximab (1000 mg × 2, 2 weeks apart) with an efficacy of 90% in the group naïve to anti-TNF and 30% in the group with prior anti-TNF failure (effectiveness defined by a 20% reduction in the Bath Ankylosing Spondylitis Disease Activity Index). Efficacy was noticeable 4 to 8 weeks after injection.^[18,19]^ Nocturne et al reported data from 8 patients treated with rituximab (3 with ankylosing spondylitis, 2 with undifferentiated spondyloarthritis and 3 with psoriatic arthritis) of whom 7 had failed anti-TNF treatment. Rituximab was effective in 2 patients.^[20]^ Rituximab has also been reported to be effective in other case reports.^[21–24]^ The reference treatments for AS remain anti-TNF agents, particularly for axial forms, and methotrexate (especially for peripheral forms with less effectiveness on axial forms). Concerning our patient, the choice of an anti-TNF agent did not seem wise due to the lack of proof of effectiveness in AAV and the axial joint damage made the choice of methotrexate less relevant, which is why we opted for rituximab.^[25,26]^

Although the beneficial effect of this treatment has been suggested in AS, the clear efficacy of rituximab in our case could support the hypothesis of a single disease explaining the clinical manifestation in our patient, that is, GPA with an atypical musculo-skeletal involvement. Besides, it confirm recent data on the effectiveness of this treatment against ENT involvement and granulomatous pseudo-tumoural forms.^[27,28]^ The rheumatic condition of our patient could also be improved initially by high-dose corticosteroid therapy, but the prolonged remission after corticosteroid therapy supports the efficacy of rituximab. One of the main limitations of this study is the relatively long delay between the onset of clinical manifestation related to spondyloarthritis and that of AAV.

## 4. Conclusion

This case illustrates the rare but possible association between inflammatory rheumatic disease and ANCA-associated vasculitis. There are some common physiopathological traits between these 2 entities that make this association probably not coincidental. In this situation, there is a need to find a treatment that is effective against both diseases, in our case rituximab. These associations of rare diseases are precisely a good way to study the common physiopathological elements and nonclassical treatments of certain diseases.

## Author contributions

**Conceptualization:** Thomas Escoda, Laurent Chiche.

**Supervision:** Benjamin Terrier, Laurent Chiche.

**Validation:** Aurélie Dehaene, Laetitia Velardocchio, Arnaud Deveze, Benjamin Terrier, Laurent Chiche.

**Writing – original draft:** Thomas Escoda.

**Writing – review & editing:** Thomas Escoda, Aurélie Dehaene, Laetitia Velardocchio, Arnaud Deveze, Benjamin Terrier, Laurent Chiche.
